# Psychological distress and mental health disparities over time between tertiary students and non-student working peers in Australia

**DOI:** 10.1007/s00127-025-02953-w

**Published:** 2025-06-19

**Authors:** Shu Mei Teo, Daniel Gan, Mengmeng Wang, Vivienne Browne, David Baker, Catherine L. Smith, Kate Filia, Eóin Killackey, Caroline X. Gao

**Affiliations:** 1 Orygen, Parkville, VIC Australia; 2 Centre for Youth Mental Health, The University of Melbourne, Parkville, VIC Australia; 3 Department of Epidemiology and Preventative Medicine, School of Public Health and Preventive Medicine, Monash University, Melbourne, VIC Australia

**Keywords:** Young people, Mental health, Psychological distress, Tertiary students

## Abstract

**Purpose:**

Tertiary students have been recognised as a high-risk population for psychological distress yet, in Australia, have been overlooked in population-level surveillance, health service provision and mental health policy. This study sought to explore trends in self-reported psychological distress and general mental health of tertiary students compared to their non-student working peers in Australia from 2007 to 2022— a timeframe which spans the pre-, mid-, and immediate-post-COVID time periods.

**Methods:**

The Household, Income and Labour Dynamics in Australia (HILDA) Survey was used as a basis for this study. Focusing on participants aged 18 to 35 from survey waves spanning 2007 to 2022 (average *n* = 4415 per year), participants were categorised into three groups: working only, tertiary students and working, and tertiary students only. Psychological distress and general mental health were measured using the Kessler-10 scale and the Mental Health Inventory-5, respectively. Weighted regression models compared outcomes between students and non-students.

**Results:**

Psychological distress and poor mental health have risen among young Australians, with the sharpest increase since 2019. Tertiary students, especially those studying only, showed significantly higher distress and poorer mental health than working peers. Although adjusting for sociodemographic and socioeconomic covariates attenuated the associations, the higher distress levels in students persisted post-2019. Loneliness, long-term disabilities, and poor general health were correlates of poor mental health across all groups.

**Conclusions:**

Findings highlight the need for targeted care models for tertiary students, including enhanced campus mental health support and screening services, financial support, social connection programs, and digital health solutions.

**Supplementary Information:**

The online version contains supplementary material available at 10.1007/s00127-025-02953-w.

## Introduction

Since the 1970s, rates of participation in tertiary education (encompassing both university and vocational education) have been increasing around the world [1]. For individuals, tertiary education may increase the prospects of obtaining a higher income, better employment security, and more opportunities for meaningful participation in the activities of society [2]. At a societal level, economies need a workforce comprised of individuals with highly specialised skills and a broad knowledge foundation to remain competitive in increasingly globalised and technology-driven job markets [3]. In Australia, 5.5 million people reported holding Bachelor (undergraduate) degree qualifications or higher in 2022—a 30.7% increase compared to 2016 [4]. Given the benefits that tertiary education brings to individuals and societies, participation rates are anticipated to continue rising in Australia and worldwide in the foreseeable future [5].

Tertiary students (individuals pursuing tertiary education), however, have been recognised as a high-risk population for psychological distress [6]. This may be due to a combination of a range of factors. Typically, tertiary education takes place during the transition from adolescence to young adulthood. This coincides with the age of onset for over 60% of known mental disorders [7]. Individuals may experience a variety of unique stressors during this critical transition time, such as living independently for the first time, adjusting to a new environment and routine, managing limited financial resources, and balancing work, social, and other commitments with the demands of tertiary education [8]. A World Health Organization (WHO)–commissioned survey of tertiary students (*N* = 20,842) from nine countries found that higher levels of stress in one or more of six aspects of everyday life—namely, financial well-being, physical well-being, family relationships, romantic relationships, school/workplace relationships and well-being of significant others–were associated with higher 12-month prevalence rates of mood-, anxiety-, or substance use-related disorders [9].

Elevated levels of psychological distress and poor mental health among tertiary students may lead to many negative consequences. These may include consequences that are detrimental to their pursuit of educational goals—such as poorer academic performance, decreased student motivation [10], and lower completion rates [11, 12]. The negative impact may also extend to other aspects of life, such as poorer physical health habits [13] and reduced social functioning [14]. Despite these findings, tertiary students have been overlooked in population-level surveillance, health service provision and mental health policy in Australia [15].

Nationwide studies that have examined longitudinal trends of mental ill-health among tertiary students have consistently reported increases in rates of both self-reported mental health difficulties and help-seeking (for example in Norway and United States; Knapstad et al., 2019; Lipson et al., 2022; Oswalt et al., 2018) [16–18]. However, to enable a nuanced understanding of the mental health impact on tertiary students, these trends need to be considered vis-à-vis their counterparts in the wider population who are not in tertiary education. In Australia, two studies have attempted this by using data from the nationwide Household, Income and Labour Dynamics in Australia (HILDA) survey [19, 20]. The earlier study found higher rates of moderate psychological distress—defined as a total score of 16–21 on the Kessler Psychological Distress Scale (K10; Kessler et al., 2002)—among tertiary students compared to their non-student counterparts, but no between-group differences in rates of high psychological distress—defined as a K10 score of 22 or greater [20]. In contrast, the more recent study found that young people who attended university generally reported *better* mental health compared to their peers who did not attend university [19]. Critically, however, individuals who pursued university education displayed poorer mental health than their counterparts at two transitional timepoints: during the final year of high school, and at the final year of undergraduate studies [19]. While the above studies have significantly contributed to our understanding of the impact of tertiary education on mental health, it is noteworthy that they were conducted prior to the coronavirus-19 pandemic (COVID-19).

It is undeniable that COVID-19 has transformed the tertiary education experience. New ways of studying and learning, characterised by increased use of remote learning environments, have been linked with poorer mental health due to limited opportunities for in-person interactions [22, 23]. While many of these digital transformations were gradually emerging in higher education before the pandemic, COVID-19 dramatically accelerated their adoption across institutions worldwide [24]. Although these strategies arose out of necessity due to physical distancing restrictions, it is anticipated that they will continue to be employed in some capacity post-pandemic [25]. Post COVID-19 research on the experience and impact of psychological distress among tertiary students is therefore needed to provide up-to-date information for guiding mental health service planning in this priority population. At present, however, existing mental health strategies in Australian universities are still largely based on outdated (pre-COVID-19) evidence [26] and approaches within vocational training providers are significantly varied depending on the size of the provider, the priorities of the leadership and the limitations of the evidence base for effective interventions [27].

This study aimed to determine whether Australian tertiary students experienced poorer mental health outcomes compared to their non-student working peers between 2007 and 2022—a timeframe which spans the pre-, mid-, and post-COVID time periods. Using data collected from the ongoing nationwide HILDA survey, the study sought to identify whether tertiary students constitute a potentially vulnerable population requiring targeted support and interventions.

## Methods

### Data source

The Household, Income and Labour Dynamics in Australia (HILDA) Survey was used as the basis for this study [28]. The HILDA survey is a nationally representative annual household-based longitudinal panel study, initiated in 2001, which collects data regarding economic and personal wellbeing, labour market dynamics and family life among Australian households. It is funded by the Australian Government and managed by the Melbourne Institute of Applied Economic and Social Research. HILDA employs a multistage stratified area sampling design. At Wave 1, approximately 7,682 households and over 13,000 individuals were recruited through a probability sample drawn from private dwellings across Australia. All household members aged 15 and over were invited to participate, and respondents, extended to include any new household members, continue to be followed over time. Additional sample top-ups were introduced in later waves (from 2011) to maintain national representativeness and improve representation of recent migrants. Data are collected annually through a combination of self-completion questionnaires and face-to-face interviews. Detailed information about the data collection methods, sampling techniques and survey items has been comprehensively documented in prior work [28, 29].

For this study, we used data from 2007 to 2022 (Waves 7–22), as 2007 marked the introduction of the Kessler Psychological Distress Scale (K10), our primary mental health outcome.

### Study population

Our study population is aged 18–35 and records from all other age groups were removed from the analysis. Tertiary students were defined as those who were enrolled in university courses (doctoral degree, master’s degree, graduate diploma, graduate certificate, or bachelor’s degree) or Vocational Education and Training (VET) courses (advanced diploma and associate degree, diploma, certificate III & IV, or certificate I & II) based on the Australian Standard Classification of Education (ASCED) codes [30]. Participants were classified into four categories: (1) Working only (employed and not a tertiary student), (2) Studying and working (employed and a tertiary student), (3) Studying only (unemployed or not in the labour force and a tertiary student), and (4) Not in education or employment (NEET, unemployed or not in the labour force and not a tertiary student). Without additional contextual information, we understand that the NEET group is likely to be heterogeneous, combining young people who are carers, job seekers or unable to work/study due to physical or mental ill-health [31], and are known to have higher prevalence of mental health conditions [32, 33] that was further supported by our data (see Results). As such, they were excluded from all regression analyses with only descriptive statistics reported.

### Measures

This study utilized two validated mental health measures available in the HILDA survey: the Kessler Psychological Distress Scale (K10) [21], and the Mental Health Inventory-5 (MHI-5) [34].

The K10, which has been collected in HILDA since 2007, is a widely used 10-item screening tool for psychological distress with strong psychometric properties including high internal consistency (Cronbach’s alpha = 0.93) [21]. K10 can be categorised into four levels: low (K10 score 10–15), moderate (K10 score 16–21), high (K10 score 22–29), and very high (K10 score 30–50) [35]. We consider a person in psychological distress if their K10 category is ‘high’ or ‘very high’. K10 scores in the upper ranges was found to be strongly associated with diagnosable mental disorders [36] and aligns with reporting approaches consistent with reports from The Australian Bureau of Statistics [37] and The Australian Institute of Health and Welfare [38]. For analyses related to K10, we used data from 8 HILDA Survey waves (waves 7,9,11,13,15,17,19, and 21).

The MHI-5 is a five-item subscale from the SF-36 designed to assess general mental health over the past month and has been shown to reliably capture general mental health, including depression and anxiety. It is also well-validated and has been widely used in population-based mental health research [39]. The total score of MHI-5 ranges between 0 and 100, with higher scores indicating better mental health. We defined poor general mental health as a score less than or equal to 52, consistent with what was used in the HILDA statistical report [40]. For analyses related to the MHI-5, we used data from 16 HILDA Survey waves (waves 7–22). Items from both the K10 and MHI-5 are listed in **Supplementary Table ****S1**.

Other potential confounding factors and variables potentially correlated with mental health outcomes are summarised in **Supplementary Table ****S2**. We adjusted for a comprehensive set of confounders in all regression models including age group, sex, culturally and linguistically diverse (CALD) background, Indigenous status, Index of Relative Socio-economic Advantage and Disadvantage (IRSAD) decile, equivalised household income, experience of financial hardship, and current marital status. Indigenous status was included as a covariate in regression models but not reported in descriptive statistics in line with our ethics approval.

We also examined a set of variables that have been shown to be associated with mental health outcomes and reflect broader domains of functioning, including loneliness or social isolation, long-term health conditions or disabilities, and poor general health [41–43]. Importantly, these variables may lie on the causal pathway between education/employment status and mental health. For this reason, we did not adjust for them in the main regression models to avoid overadjustment bias. Instead, we present descriptive comparisons to highlight the extent of co-occurring challenges among those experiencing poor mental health.

### Statistical analysis

#### Overview and weighting strategy

All analyses were conducted using **R** version 4.2.1 (2022-06-23). To obtain estimates most relevant to the Australian community at each time point, we conducted cross-sectional analyses using the self-completion questionnaire (SCQ) responding person weight (cross-section population weight rescaled to sum to the number of responding persons in the relevant wave) in all analyses. The analyses in this paper were not pre-registered.

#### Descriptive analysis

Simple descriptive statistics were used to describe participants’ demographics and their mental health and wellbeing. Graphical visualisations demonstrated the mental health and wellbeing trends over time.

#### Assessing differences in mental health between tertiary students and working peers

Our main study aim was to establish whether tertiary students had poorer mental health compared to their working non-student peers, and whether this relationship differs depending on whether students are studying only or studying while working. To answer this, we ran weighted linear or logistic regression models with mental health outcomes as dependent variables (continuous K10 scores, a binary indicator for high to very high psychological distress using K10 ≥ 22, continuous MHI-5 scores, and a binary indicator for poor general mental health using MHI-5 score ≤ 52). For each survey year, we compared (1) all tertiary students vs. working only young people, (2) studying only young people vs. working only young people, and (3) studying and working young people vs. working only young people.

#### Exploring variation by age group and sex

Additionally, to explore whether these associations varied by age group or sex, we ran age group and sex stratified models and used random-effects meta-regression (REML method via R function *metafor::rma*) to test for effect modification.

#### Pooling estimates across the survey years

To obtain an overall estimate across all survey years, we pooled the year-specific estimates using random effects meta-analysis (REML method via R function *metafor::rma*).

#### Handling missing data and covariate adjustment

Missing data were addressed using multiple imputation by chained random forest [20 imputed datasets using R function *missRanger;* Mayer [44] and pooled using Rubin’s rules [45]. In the adjusted models, the following variables were included: age, sex, IRSAD, equivalised household income, financial hardship, CALD status, Indigenous status, and marital status (see **Supplementary Table ****S1** for variable definitions). Covariates for adjustment were selected by building a directed acyclic graph (DAG) based on expert knowledge and literature review (**Supplementary Figure ****S1**). The *dagitty* R package was used to check the implied conditional independencies of the postulated DAG using chi-squared tests. We performed tests for conditional independencies of the variables separately for each imputed dataset and each year. We then averaged the test statistics, including the root mean square error of approximation (RMSEA), across all years and imputed datasets (**Supplementary Table S3)**.

## Results

### Population characteristics

Within our population of interest (18–35-year-olds), participants were classified into the following three groups: (1) Working only, (2) Studying and working, and (3) Studying only. Proportions of participants across the groups remained relatively stable over this period, with an average of 70% in the working only group, 22% in the studying and working group, and 8% in the studying only group (see **Supplementary Table S4** for the sample sizes and weighted proportion of participants across the groups each year).

Descriptive characteristics of the 2021 sample are presented in Table 1 (see **Supplementary Tables S5-S12** for the summary characteristics of participants including those who are NEET, and for the other years). Compared to our control group (working only young people), NEET young people had worse mental health throughout the years. For example, in 2021, 47% of NEET young people reported high to very high psychological distress and 34% with poor mental health on the MHI-5 scale, compared to 30% and 21% respectively for the working only group (see **Supplementary Tables S5-S12** for other years).

**Table 1:** Participant characteristics in 2021 (weighted)

	Working only	Studying and working	Studying only	Overall (*N* = 3874)
Age group				
18–25	817 (30.7%)	574 (64.2%)	219 (69.2%)	1610 (41.5%)
26–35	1847 (69.3%)	319 (35.8%)	97 (30.8%)	2264 (58.5%)
Sex				
Male	1383 (51.9%)	435 (48.7%)	147 (46.4%)	1965 (50.7%)
Female	1281 (48.1%)	458 (51.3%)	169 (53.6%)	1909 (49.3%)
IRSAD, (mean, SD)	5.9 (2.8)	6.3 (2.8)	6.1 (2.9)	6.0 (2.8)
Equivalised household income, (mean, SD)	78566.8 (34956.7)	79938.9 (56045.6)	64827.4 (67233.1)	77761.8 (44089.1)
Financial Hardship	471 (18.5%)	182 (21.7%)	106 (34.6%)	759 (20.6%)
CALD	437 (16.4%)	176 (19.7%)	106 (33.5%)	719 (18.5%)
Marital status: Married	1444 (54.2%)	291 (32.6%)	57 (18.0%)	1792 (46.3%)
MHI-5				
Mean (SD)	68.2 (17.3)	64.5 (18.1)	58.3 (20.0)	66.5 (18.0)
Median (Q1, Q3)	72.0 (56.0, 80.0)	68.0 (52.0, 80.0)	64.0 (44.0, 72.0)	68.8 (56.0, 80.0)
MHI-5 < 52	553 (20.9%)	249 (28.0%)	116 (37.4%)	918 (23.9%)
K10 score				
Mean (SD)	19.1 (7.6)	20.7 (7.9)	22.8 (8.8)	19.8 (7.8)
Median (Q1, Q3)	17.0 (13.0, 23.0)	19.0 (14.0, 27.0)	22.0 (15.0, 29.0)	18.0 (13.0, 25.0)
K10 ≥ 22	779 (29.8%)	338 (39.0%)	167 (54.1%)	1284 (33.9%)

**Fig. 1:**
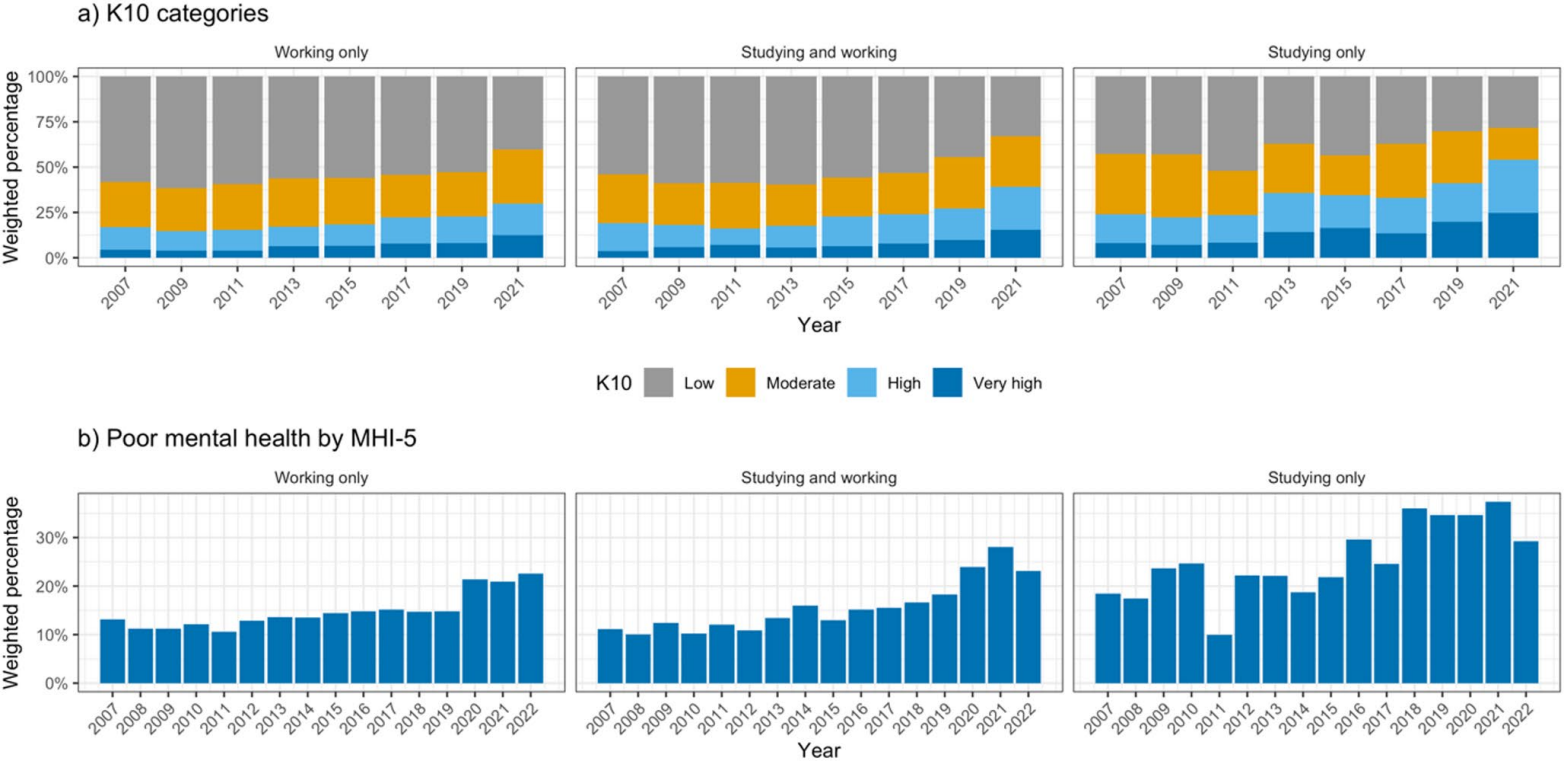
Weighted percentage of (**a**) K10 categories and (**b**) poor mental health by MHI-5 from 2007–2022, by tertiary student and employment status

Working only young people were more often in the older age group (70% 26–35-year-olds, compared to 36% studying and working young people, and 31% studying only young people in 2021, Table 1). Studying only young people were more often culturally and linguistically diverse (CALD, 34%, compared to 16% and 20% for working only young people and working and studying young people respectively in 2021), slightly more often female (54% compared to 48% and 51% respectively as above), had lower equivalised family income (mean of 64.8 K compared to 78.6 K and 79.9 K respectively as above), and more experienced financial hardship (35%, compared to 19% and 22% respectively as above). For all groups, both K10 and MHI-5 mental health scores show worsening mental health over time, particularly from 2019 onwards (Figs. 1 and 36% high to very high psychological distress in 2021, up from 27% in 2019, and 25% with poor mental health in 2022, up from 19% in 2019).

### Association between tertiary student status and mental well-being

**Fig. 2:**
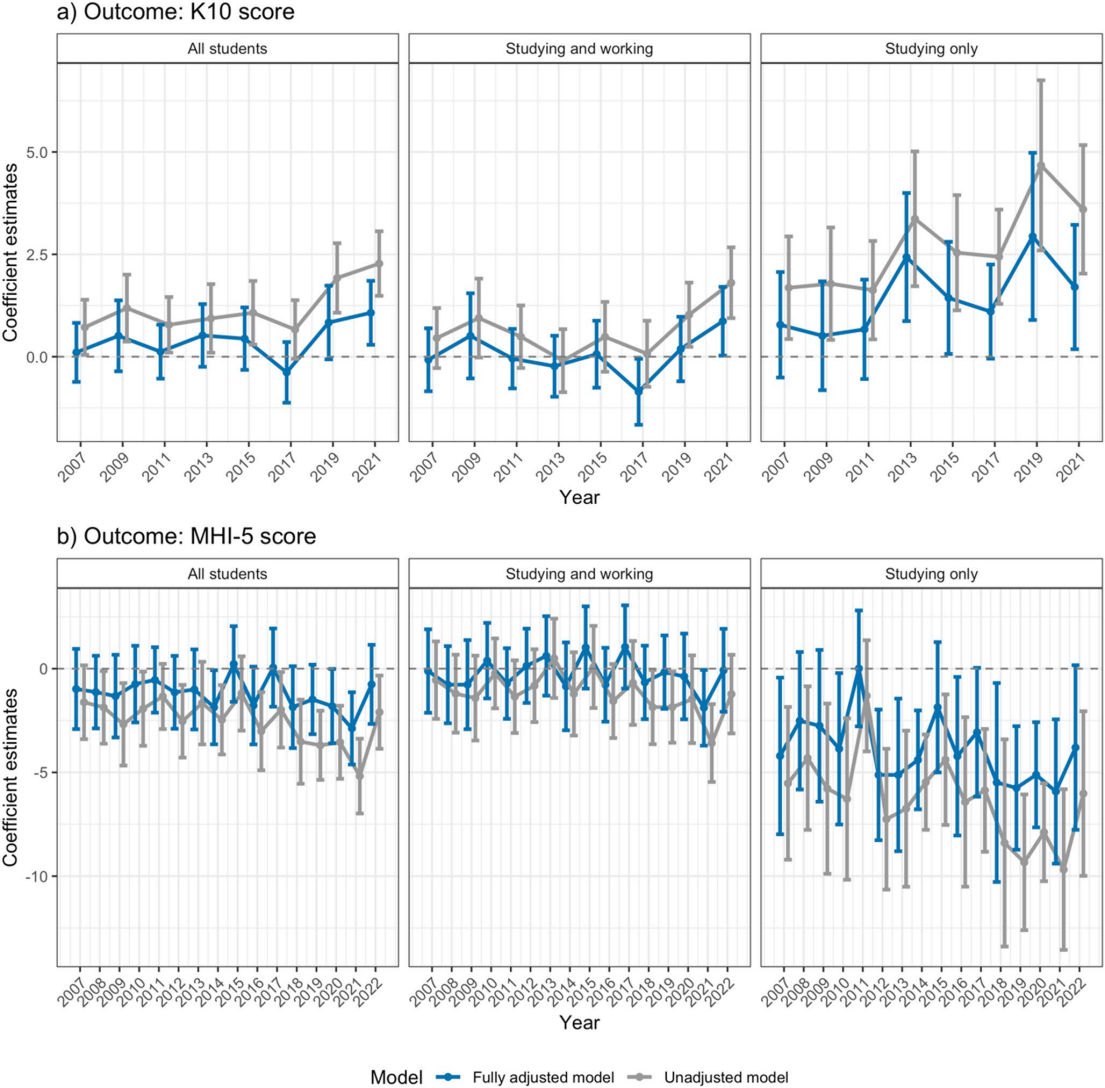
Beta coefficients and 95% confidence intervals from weighted linear regression of (**a**) K10 scores, (**b**) MHI-5 scores against tertiary student and employment status. (Left) compares all students with working only young people, (Middle) compares studying and working young people with working only young people, (Right) compares studying only young people with working only young people. The adjusted model included the following covariates: age, sex, IRSAD, equivalised household income, experience of financial hardship,CALD status, Indigenous status, and marital status

Tertiary students have higher psychological distress and lower mental wellbeing compared to their working counterparts, especially in recent years from 2019 to 2022 (b[95% CI]: 2.28[1.49,3.06] and − 5.18[-6.98,-3.38] for K10 and MHI-5 in 2021 respectively in the unadjusted models comparing all students vs. working only young people, Fig. 2). The studying only group conferred the highest risk, followed by the studying and working group. Additionally adjusting for possible confounders attenuated the associations, though they remained significant in 2021.

**Fig. 3:**
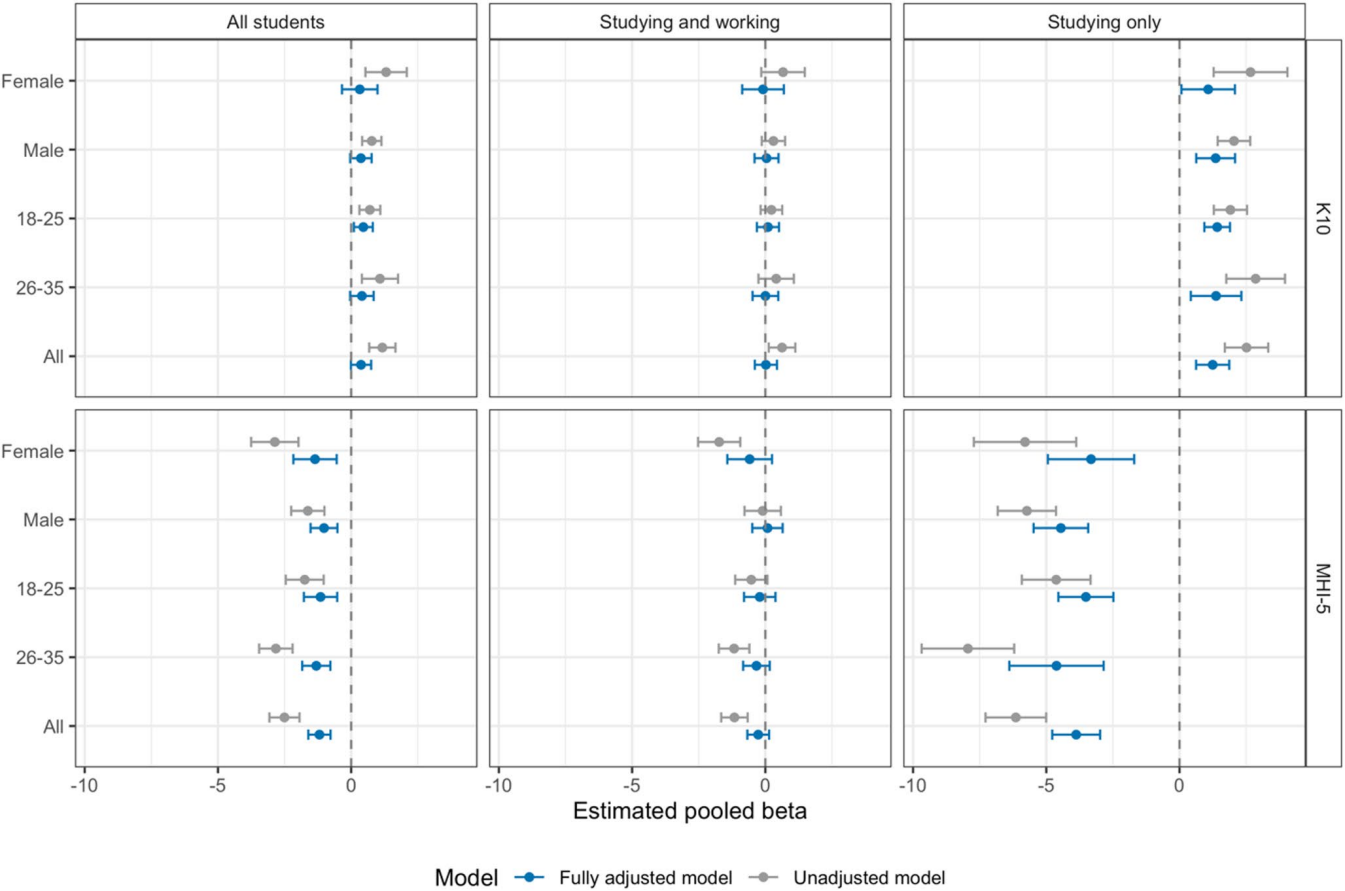
Average (across all years) beta coefficients and 95% confidence intervals of weighted linear regression of (top panels) K10 scores and (bottom panels) MHI-5 scores against tertiary student and employment status. Coefficients were averaged using a random effects meta-analysis with the REML method

There was no evidence of effect modification by sex or age group, i.e. the effect estimates were fairly consistent when stratified by sex or age group (Fig. 3). However, the associations seem slightly stronger among females compared to males as well as among 26–35 year olds compared with 18–25 year olds.

We also ran weighted logistic regression of binary indicators of poor mental health (K10 ≥ 22, MHI-5 ≤ 52) and obtained similar conclusions (OR [95% CI]: 1.81[1.46,2.24] and 1.64[1.30,2.07] for K10 and MHI-5 in 2021 respectively in the unadjusted models, **Supplementary Figure ****S2**, see **Supplementary Figure S3** for the stratified and pooled results).

### Correlates of poor mental health

**Fig. 4:**
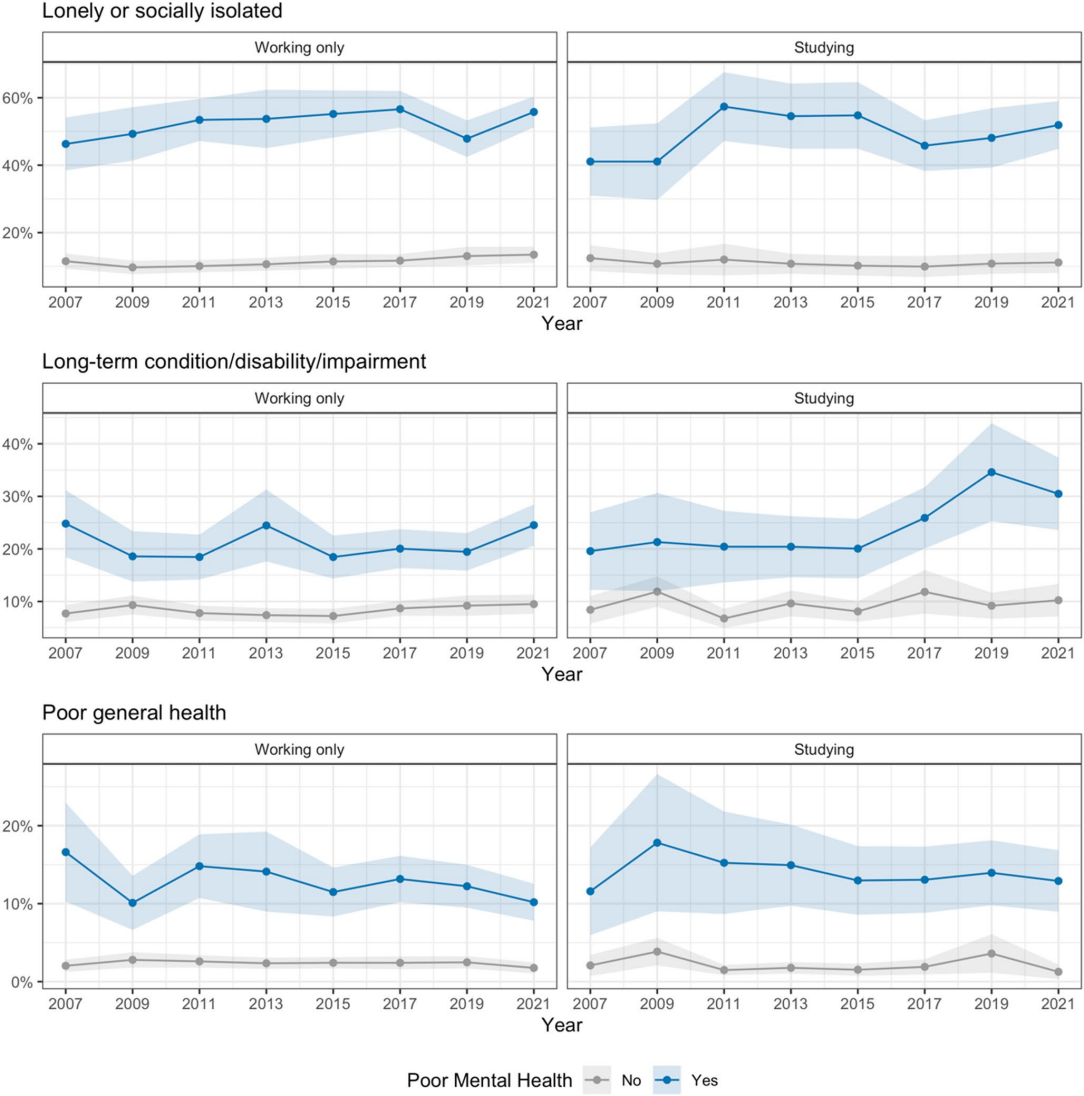
Percentages and corresponding 95% confidence intervals of young people who are (top panel) lonely or socially isolated, (middle panel) have a long-term condition, and (bottom panel) are in poor general health among those with poor mental health (blue) and without poor mental health (grey). (left panels) shows the working only group, and (right panels) shows the tertiary students

Finally, we were interested in correlates of poor mental health (defined here as those who were experiencing high to very high psychological distress, or poor mental health on the MHI-scale), such as experiencing loneliness or social isolation, having a long-term health condition, disability or impairment that restricts everyday activity, and being in poor general health. The percentage of people with poor mental health has increased over the years, from 20% (95% CI: 17-22%) in 2007 to 33% (95% CI: 30-35%) in 2021 for the working only young people, and 23% (95% CI: 19-27%) in 2007 to 47% (95% CI: 42-51%) in 2021 for tertiary students. Young people experiencing poor mental health showed substantially higher rates of additional challenges compared to those with good mental health, regardless of study status. Among those with poor mental health, 50.1% reported experiencing loneliness or social isolation, compared to 11.2% among those without poor mental health (averaged across the years). Similarly, long-term disability/impairment was reported by 22.2% of those with poor mental health versus 8.7% of those without, and poor general health reported by 13.3% of those with poor mental health versus 2.3% of those without (Fig. 4).

## Discussion

This study is the most recent evaluation of tertiary student mental health in Australia, providing a comprehensive overview of mental health trends among young people aged 18–35 over a period of more than 15 years, from 2007 to 2022. This period spans before, during, and after the COVID-19 pandemic. The results indicate that psychological distress and mental wellbeing in general is worsening over time among young people, a trend that is consistent with previous research findings [46, 47]. However, our findings reveal important nuances that warrant careful interpretation.

When examining tertiary students as a whole, we observed that their mental health was largely comparable to their working peers across most of the study period, with some elevation in poor mental health in more recent years. This pattern was primarily driven by the subgroup of students who were studying only, without concurrent employment, suggesting a complex relationship between education, employment, and mental health. The observed patterns were remarkably consistent across both mental health measures utilized in this study– the K10 and MHI-5 scales– which substantiates the robustness of our findings.

Several explanations may account for these patterns. Working individuals may experience more structured routines, better social engagement, and financial stability which can serve as protective factors for mental health [48]. Conversely, students who are not working may face greater financial strain or may have pre-existing conditions that prevent them from balancing work and study simultaneously [49]. In summary, our findings align with research on the protective effects of employment on mental health [50, 51]. The finding that tertiary students in paid employment had slightly better mental health compared to those studying only also aligns with previous research highlighting the potential benefits of certain job characteristics (e.g. jobs related to field of study and those offering autonomy) for student wellbeing [52, 53]. However, further longitudinal research is needed to establish directional relationships. Specifically, it is crucial to acknowledge that the relationship between employment and student wellbeing is complex and not uniformly positive. Factors such as high job demands and excessive working hours have been shown to negatively impact student wellbeing [52, 53], underscoring the nuanced nature of this relationship.

The Australian context of this study also deserves specific consideration when generalizing findings internationally. Combined work and study are relatively common in Australia, with approximately 70% of students engaging in paid employment while studying. This differs from some international contexts where studying without employment is more normative, or where institutional structures (such as different funding models) may alter the student experience. The protective effect of employment we observed may therefore be particularly relevant in systems similar to Australia’s but might manifest differently in countries with different educational and employment structures.

An important insight from the inclusion of recent data was that the disparity in psychological distress between students and non-students widened in 2019—before the pandemic took place. This finding suggests that a trend of worsening psychological distress among tertiary students in Australia had been forming prior to the pandemic but continued to worsen. In 2021, nearly half of our tertiary student sample experienced poor mental health. These findings highlight the critical need for targeted and scalable mental health interventions for tertiary students to mitigate this concerning trend. Traditional in-person counselling services, while valuable, may not be sufficient to address the widespread and growing mental health needs of this population. To effectively manage the high demand, scalable care models that can reach a larger number of students are essential. One emerging possibility is the use of technology-supported mental health platforms designed to provide users with meaningful supportive interaction with healthcare professionals and peers, as well as evidence-based skills and knowledge for self-management of mental health difficulties. In Australia, evidence of the use of these platforms for improving social and psychological wellbeing among young people is nascent but promising [54].

In the earlier years of the study, roughly 2007 to 2017, adjusting for sociodemographic and socioeconomic factors significantly attenuated the associations between student status and poorer mental health. This attenuation suggests that a substantial portion of the observed differences in mental health outcomes can be explained by these confounding variables. For example, females and individuals experiencing financial hardship are associated with higher levels of psychological distress, and not being married can be associated with lower social support and increased stress.

Further, our findings also highlighted the importance of other indicators of social exclusion such as loneliness and lack of social support towards contributing to greater psychological distress in the overall sample, regardless of tertiary education status. This is congruent with past research demonstrating that indicators of social exclusion are risk factors for psychological distress among young people [55–57]. In the context of tertiary education, it has been noted that the impact of low perceived social support (such as self-reported loneliness or social isolation) on mental ill-health may be amplified through reduced opportunities for in-person social interaction that occur as a by-product of the increased use of distance learning methods [58].

In summary, this study highlights important mental health challenges facing certain groups of tertiary students in Australia, particularly those studying without concurrent employment. While the mental health gap between tertiary students and their working peers has widened in recent years, this appears to be driven by specific subgroups and contexts rather than representing a universal crisis in tertiary education. Targeted approaches that address financial security, social connection, and the specific needs of vulnerable student populations may be most effective in supporting student wellbeing within the Australian tertiary education system.

### Policy implications

The research findings have several implications for policy makers across health promotion, mental health and tertiary education. That student mental health has worsened in Australia in recent years requires a renewal of focus and efforts to both: (a) increase our understanding of the risk and protective factors for tertiary student mental health and (b) deliver evidence-based, holistic responses to student wellbeing, both within tertiary education settings and linked with community and population level mental health responses.

For the former, there is a need to undertake further research on the specific experiences of tertiary education that may contribute to both positive wellbeing and poor mental health. Some contributing factors to poor mental health and wellbeing for tertiary students will exist outside the influence of education settings. However, understanding what specific attributes or experiences of study at this level contribute to psychological distress or reduced wellbeing will assist policy makers and tertiary education leaders, administrators and staff to make modifications to the educational environment, curriculum or service provision to better respond. In addition, as found through this research, mental health issues across the student population can vary over time. There is a need for greater regularity in data collection across tertiary student populations– where possible using existing student experience surveys or data collection tools to include measures of mental health and wellbeing.

To the second policy implication for more evidence-based and effective holistic responses to student mental health and wellbeing, existing frameworks and guidelines developed in the UK [59], Australia [26] and Canada [60] all acknowledged the need for whole of institution responses to mental health and wellbeing and approaches across promotion, prevention, early intervention and treatment. Further support may be needed for implementation, including funding and/or more requirements directed by education policy makers that universities are to implement and report on progress. Particular areas of focus as identified through the correlates in this research include increasing financial aid/scholarships to alleviate financial pressures for tertiary students which may be contributing to psychological distress; reinvigoration of activities that enhance student connection and inclusion on tertiary education campuses and online including student societies, interest groups, clubs and buddy/mentoring programs; enhanced mental health services in tertiary education that provide free care and support and which also leverage on digital platforms to increase opportunities to access both on and off campus.

### Strengths, limitations, and future directions

This study leverages data from the Household, Income and Labour Dynamics in Australia (HILDA) Survey, a household-based panel survey that annually examines a wide range of social, economic and health questions. Using data from 16 waves of the HILDA survey has enabled us to examine time trends of prevalence and associations from 2007 to 2022. We treated each wave as a repeated cross-sectional sample, applying survey weights to produce estimates representative of the Australian population at each timepoint. This cross-sectional approach allowed us to maximise sample size at each wave and focus specifically on the comparison of concurrent mental health states between tertiary students and non-studying working peers, rather than developmental trajectories over time. However, an important limitation of this approach is that we could not adjust for earlier risk factors or account for the longitudinal dynamics of participants joining, remaining, or leaving the cohort. Future research using longitudinal modelling to trace changes in mental wellbeing throughout different educational engagement and employment stages would be valuable. Such analyses could help disentangle whether observed mental health differences are attributable to pre-existing risk factors or to the educational and employment experiences themselves.

Another important limitation of our analytical approach is that we included all covariates simultaneously in our models, which, while comprehensive for confounding control, does not allow us to assess the relative contribution of individual factors to the attenuation of associations. Additional formal mediation analyses could provide insights into which factor most strongly contribute to the observed associations between education/employment status and mental health.

The study also relied on the Kessler Psychological Distress scale and the Mental Health Inventory-5 as measures of psychological distress and mental health. While these tools are widely recognized and validated for screening of general psychological distress and offering a broad overview of overall mental well-being, they do not provide clinical diagnoses and may not encompass the entire spectrum of mental health disorders. This means our findings may not fully capture the complexity and nuances of different mental health issues among tertiary students.

In addition, while it was beyond the scope of this study, future research could also examine the associations between mental well-being and different university and VET courses, as well as the impact of poor mental health on academic performance. Understanding how specific academic environments and disciplines impact mental health can inform tailored interventions.

In conclusion, this study has provided valuable insights into the mental health trends of young people, particularly tertiary students, over a significant period, up to 2022. It is crucial to continue monitoring the mental health of young people in the coming years to assess recovery trends and resilience post-pandemic, including understanding how tertiary students are adapting to new educational models and the long-term effects of pandemic-related disruptions.

## Electronic supplementary material

Below is the link to the electronic supplementary material.

Supplementary Material 1

## Data Availability

Research data is available at https://dataverse.ada.edu.au/dataverse/DSSLongitudinalStudies. All analysis code is available at https://osf.io/d69wn/?view_only=efe32c0309e7455699caaa4459fe8066.
