# Supplementary material for: Psychological distress and mental health disparities over time between tertiary students and non-student working peers in Australia
Source: Soc Psychiatry Psychiatr Epidemiol. 2025 Jun 19;60(12):2835–47. doi: 10.1007/s00127-025-02953-w (PMC12594646; doi:10.1007/s00127-025-02953-w)
Supplement: Supplementary file 1 — Supplementary Material 1 [file 127_2025_2953_MOESM1_ESM.docx]

**Supplementary materials**

**
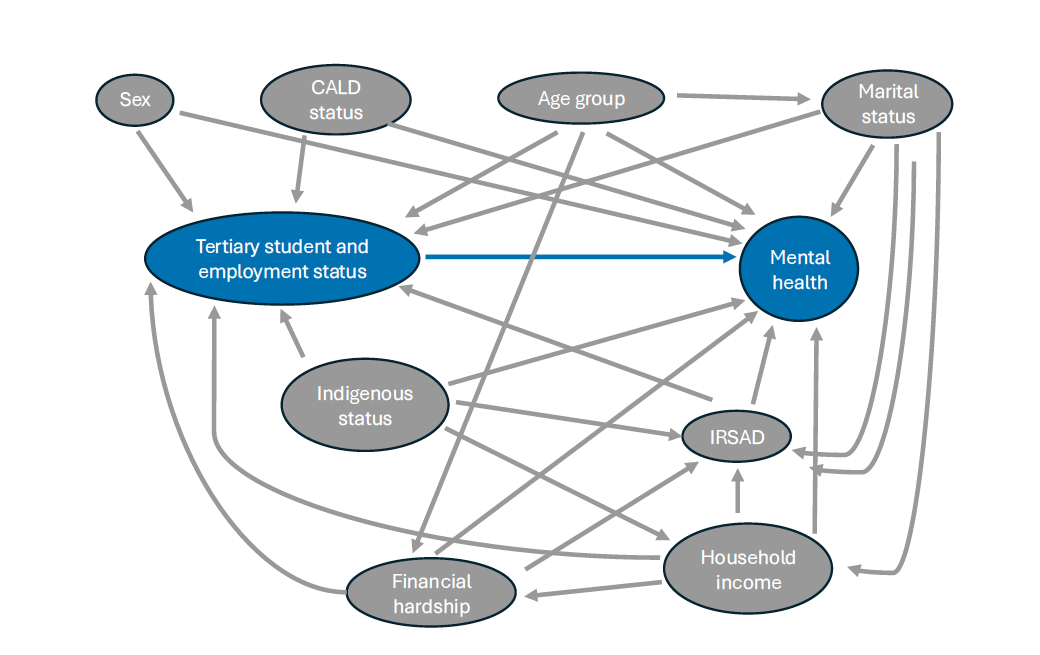
**

**Figure S1:** Directed Acyclic Graph (DAG) illustrating the relationships between psychological distress/mental wellbeing (outcome) and tertiary student and employment status of young people (exposure), including possible factors that influence the exposure and/or outcome.


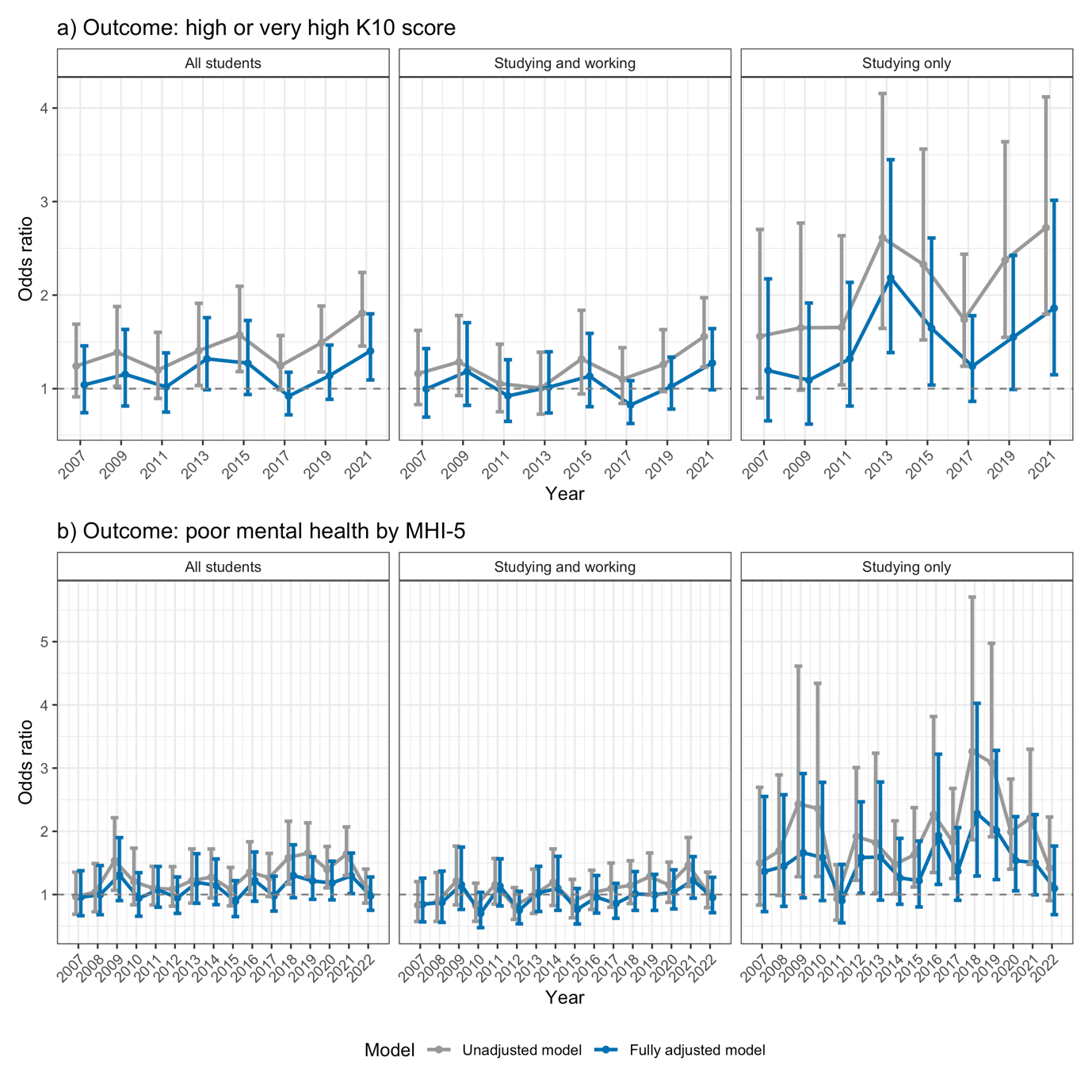


**Figure S2**: Odds ratios and 95% confidence intervals from weighted logistic regression of (a) K10 ≥ 22, (b) MHI-5 ≤ 52 against tertiary student and employment status. (Left) compares all students with working only young people, (Middle) compares studying and working young people with working only young people, (Right) compares studying only young people with working only young people. The adjusted model included the following covariates: age, sex, IRSAD, equivalised household income, experience of financial hardship, CALD status, Aboriginal status, and marital status. Corresponds to Figure 2 in main manuscript.


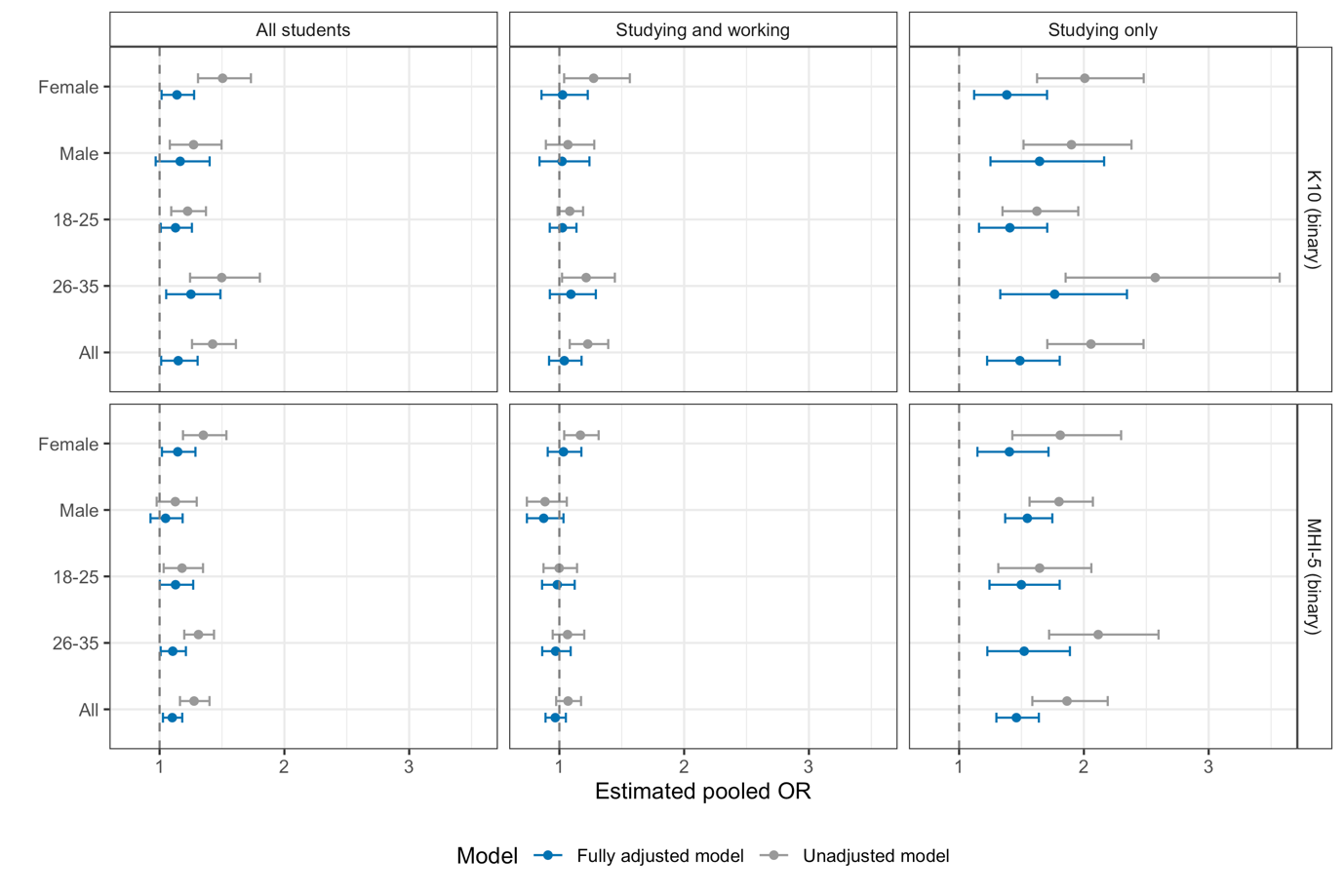


**Figure S3**: Average (across all years) odds ratios and 95% confidence intervals of weighted logistic regression of (top panels) K10 ≥ 22, (bottom panels) MHI-5 ≤ 52 against tertiary student and employment status. Coefficients were averaged using a random effects meta-analysis with the REML method. Corresponds to Figure 3 in main manuscript.

**Table S1. Items from K10 and MHI-5**

| **Items from K10 (In the last four weeks, about how often did you feel …)** | **Items from MHI-5 (How much of time during the past 4 weeks have you …)** |
| --- | --- |
| 1. Tired out for no reason 2. Nervous 3. So nervous that nothing could calm you down 4. Hopeless 5. Restless or fidgety 6. So restless that you could not sit still 7. Depressed 8. That everything was an effort 9. So sad that nothing could cheer you up 10. Worthless | 1. Been a nervous person 2. Felt so down in the dumps that nothing could cheer you up 3. Felt calm and peaceful 4. Felt down 5. Been a happy person |

**Table S2. Variables used in this study**

| Variable | Definition |
| --- | --- |
| Age group | 18–25 or 26–35 |
| Sex | Male or Female |
| Culturally and linguistically diverse (CALD) | Speak a language other than English or Country of birth not Australia/Main English speaking |
| Aboriginal or Torres Straits islander | Aboriginal and/or Torres Strait Islander and born in Australia |
| Index of Relative Socio-economic Advantage and Disadvantage (IRSAD) | Decile of ABS 2011 IRSAD based on household statistical local area |
| Equivalised household income | Household financial year disposable regular income divided by the square root of number of in-scope persons in household |
| Financial hardship | Yes if the respondent answered positively to “Since January [insert year of study] did any of the following happen to you because of a shortage of money?”: “Could not pay electricity, gas or telephone bills on time”, “Asked for financial help from friends or family”, “Could not pay the mortgage or rent on time”, “Pawned or sold something”, “Was unable to heat home”, “Went without meals”, or “Asked for help from welfare or community organizations”. |
| Current marital status | Legally married or de facto are classified as “married” |
| Disability/impairment | Answer to the question: Do you have any long-term health condition, impairment or disability (such as these) that restrict you in your everyday activities, and has lasted or is likely to last, for 6 months or more? “Yes” or “No” were accepted. “Refused” or “Don’t know” were treated as missing. |
| Lonely or socially isolated | Those in the lowest quartile (the lowest 25%) based on the sum of 10-item social support (seven-point scale, 1: strongly disagree, 7: strongly agree), ranges from 10 to 70 (scales are inverted for five items).(Scutella, 2009)   1. People don’t come to visit me as often as I would like *(scale inverted)* 2. I often need help from other people but can’t get it *(scale inverted)* 3. I seem to have a lot of friends 4. I don’t have anyone I can confide in *(scale inverted)* 5. I have no one to lean on in times of trouble *(scale inverted)* 6. There is someone who can always cheer me up when I’m down 7. I often feel very lonely *(scale inverted)* 8. I enjoy the time I spend with the people who are important to me 9. When something’s on my mind, just talking with the people I know can make me feel better 10. When I need someone to help me out, I can usually find someone |
| Poor general health | Score less than or equal to 37 on the SF-36 general health scale(Wilkins et al., 2024) |

**Table S3:** Chi-Square Test Results for Conditional Independence Assumptions in the Directed Acyclic Graph (see Figure S1)

| Assumptions tested | In K10 model | | | In MHI-5 model | | |
| --- | --- | --- | --- | --- | --- | --- |
|  | rmsea | x2 | p.value | rmsea | x2 | p.value |
| agegroup \|\| household income \| marital status | 0.066 | 20.252 | 0.005 | 0.066 | 33.741 | 0.003 |
| agegroup \|\| CALD status | 0.052 | 4.022 | 0.019 | 0.055 | 4.005 | 0.013 |
| agegroup \|\| irsad \| financial hardship, household income, marital status | 0.051 | 17.758 | 0.009 | 0.052 | 12.568 | 0.021 |
| CALD status \|\| financial hardship | 0.046 | 0.004 | 0.113 | 0.042 | 0.736 | 0.131 |
| ATSI status \|\| CALD status | 0.044 | 9.261 | 0.017 | 0.045 | 15.492 | 0.031 |
| ATSI status \|\| financial hardship \| household income, marital status | 0.044 | 6.569 | 0.039 | 0.036 | 9.600 | 0.093 |
| Sex \|\| agegroup | 0.033 | 2.223 | 0.053 | 0.024 | 0.001 | 0.175 |
| Sex \|\| financial hardship | 0.030 | 3.338 | 0.110 | 0.030 | 7.734 | 0.118 |
| agegroup \|\| ATSI status | 0.023 | 1.564 | 0.112 | 0.024 | 1.466 | 0.098 |
| CALD status \|\| household income | 0.021 | 0.146 | 0.296 | 0.015 | 1.211 | 0.327 |
| CALD status \|\| marital status | 0.014 | 4.705 | 0.407 | 0.008 | 1.541 | 0.442 |
| CALD status \|\| irsad | 0.012 | 4.418 | 0.417 | 0.013 | 0.290 | 0.396 |
| Sex \|\| ATSI status | 0.005 | 0.028 | 0.533 | 0.005 | 0.121 | 0.535 |
| Sex \|\| irsad | 0.004 | 0.318 | 0.451 | 0.002 | 0.082 | 0.520 |
| Sex \|\| household income | 0.003 | 1.121 | 0.529 | 0.003 | 1.823 | 0.480 |
| ATSI status \|\| marital status | 0.002 | 0.856 | 0.513 | 0.003 | 0.412 | 0.509 |
| Sex \|\| marital status | 0.001 | 0.477 | 0.492 | 0.002 | 1.078 | 0.478 |
| Sex \|\| CALD status | 0.000 | 0.390 | 0.544 | 0.002 | 0.310 | 0.528 |

Notes: || = “conditional independent”, | = “given”, rmsea = Root Mean Square Error of Approximation, X^2^ = chi-square statistic. Rmsea < 0.05 indicates a close fit of the model and supports the conditional independence assumption, rmsea of 0.05 to 0.08 indicates a reasonable fit. All statistics were averaged across all imputed datasets and years.

**Table S4:** Sample sizes and proportions for the groups of interest, from 2007 to 2022.

| **Year** | **Working only** | **Studying and working** | **Studying only** | **NEET** |
| --- | --- | --- | --- | --- |
| 2007 | 2319 (76.6%) | 558 (18.4%) | 152 (5.0%) | 517 |
| 2008 | 2227 (73.7%) | 627 (20.7%) | 169 (5.6%) | 485 |
| 2009 | 2137 (70.7%) | 629 (20.8%) | 256 (8.5%) | 596 |
| 2010 | 2293 (70.6%) | 686 (21.1%) | 268 (8.3%) | 537 |
| 2011 | 2662 (65.9%) | 1026 (25.4%) | 353 (8.7%) | 790 |
| 2012 | 2715 (68.0%) | 937 (23.5%) | 340 (8.5%) | 823 |
| 2013 | 2752 (67.9%) | 913 (22.5%) | 387 (9.6%) | 819 |
| 2014 | 2795 (69.2%) | 877 (21.7%) | 364 (9.0%) | 872 |
| 2015 | 2817 (67.6%) | 964 (23.1%) | 386 (9.3%) | 750 |
| 2016 | 2991 (68.7%) | 954 (21.9%) | 407 (9.3%) | 799 |
| 2017 | 3038 (69.8%) | 984 (22.6%) | 328 (7.5%) | 766 |
| 2018 | 3089 (72.0%) | 897 (20.9%) | 306 (7.1%) | 757 |
| 2019 | 3072 (71.9%) | 905 (21.2%) | 296 (6.9%) | 824 |
| 2020 | 2788 (68.9%) | 851 (21.0%) | 407 (10.1%) | 805 |
| 2021 | 2665 (68.8%) | 893 (23.1%) | 316 (8.2%) | 681 |
| 2022 | 2780 (71.9%) | 890 (23.0%) | 197 (5.1%) | 599 |

Note: All statistics reported were weighted, proportions do not include NEET. NEET = Not in education, employment, or training.

**Table S5:** Participant characteristics in 2021, including NEET. Corresponds to Table 1 in main manuscript.

|  | Working only | Studying and working | Studying only | NEET |
| --- | --- | --- | --- | --- |
| **Age group** |  |  |  |  |
| 18-25 | 817 (30.7%) | 574 (64.2%) | 219 (69.2%) | 254 (37.3%) |
| 26-35 | 1847 (69.3%) | 319 (35.8%) | 97 (30.8%) | 427 (62.7%) |
| **Sex** |  |  |  |  |
| Male | 1383 (51.9%) | 435 (48.7%) | 147 (46.4%) | 311 (45.6%) |
| Female | 1281 (48.1%) | 458 (51.3%) | 169 (53.6%) | 370 (54.4%) |
| **IRSAD**, (mean, SD) | 5.9 (2.8) | 6.3 (2.8) | 6.1 (2.9) | 4.5 (2.8) |
| **Equivalised household income,** (mean, SD) | 78566.8 (34956.7) | 79938.9 (56045.6) | 64827.4 (67233.1) | 50545.7 (25956.9) |
| **Financial Hardship** | 471 (18.5%) | 182 (21.7%) | 106 (34.6%) | 246 (38.0%) |
| **CALD** | 437 (16.4%) | 176 (19.7%) | 106 (33.5%) | 118 (17.3%) |
| **Marital status: Married** | 1444 (54.2%) | 291 (32.6%) | 57 (18.0%) | 263 (38.6%) |
| **MHI-5** |  |  |  |  |
| Mean (SD) | 68.2 (17.3) | 64.5 (18.1) | 58.3 (20.0) | 62.3 (20.5) |
| Median (Q1, Q3) | 72.0 (56.0, 80.0) | 68.0 (52.0, 80.0) | 64.0 (44.0, 72.0) | 64.0 (48.0, 80.0) |
| MHI-5 < 52 | 553 (20.9%) | 249 (28.0%) | 116 (37.4%) | 231 (34.0%) |
| **K10 score** |  |  |  |  |
| Mean (SD) | 19.1 (7.6) | 20.7 (7.9) | 22.8 (8.8) | 21.9 (9.2) |
| Median (Q1, Q3) | 17.0 (13.0, 23.0) | 19.0 (14.0, 27.0) | 22.0 (15.0, 29.0) | 20.0 (14.0, 29.0) |
| K10 ≥ 22 | 779 (29.8%) | 338 (39.0%) | 167 (54.1%) | 310 (46.5%) |

**Table S6:** Participant characteristics in 2019

|  | Working only | Studying and working | Studying only | NEET |
| --- | --- | --- | --- | --- |
| **Age group** |  |  |  |  |
| 18-25 | 1004 (32.7%) | 587 (64.8%) | 214 (72.3%) | 302 (36.6%) |
| 26-35 | 2068 (67.3%) | 318 (35.2%) | 82 (27.7%) | 522 (63.4%) |
| **Sex** |  |  |  |  |
| Male | 1679 (54.6%) | 386 (42.7%) | 126 (42.6%) | 355 (43.1%) |
| Female | 1394 (45.4%) | 519 (57.3%) | 170 (57.4%) | 469 (56.9%) |
| **IRSAD**, (mean, SD) | 6.0 (2.8) | 6.2 (2.8) | 5.7 (3.1) | 4.7 (2.8) |
| **Equivalised household income,** (mean, SD) | 73858.3 (40998.2) | 74751.4 (43144.2) | 57578.5 (67538.5) | 48477.0 (31653.2) |
| **Financial Hardship** | 610 (20.2%) | 214 (24.2%) | 113 (40.0%) | 309 (38.9%) |
| **CALD** | 674 (21.9%) | 151 (16.6%) | 67 (22.7%) | 184 (22.3%) |
| **Marital status: Married** | 1629 (53.0%) | 277 (30.6%) | 80 (27.1%) | 352 (42.7%) |
| **MHI-5** |  |  |  |  |
| Mean (SD) | 71.2 (17.0) | 69.4 (17.1) | 62.0 (20.4) | 64.3 (20.8) |
| Median (Q1, Q3) | 76.0 (60.0, 84.0) | 72.0 (60.0, 80.0) | 64.0 (52.0, 80.0) | 68.0 (52.0, 80.0) |
| MHI-5 < 52 | 451 (14.8%) | 164 (18.3%) | 102 (34.6%) | 247 (30.0%) |
| **K10 score** |  |  |  |  |
| Mean (SD) | 17.3 (7.0) | 18.3 (7.2) | 22.0 (9.5) | 20.5 (9.1) |
| Median (Q1, Q3) | 15.0 (12.0, 21.0) | 16.0 (13.0, 23.0) | 21.0 (14.0, 27.0) | 18.0 (13.0, 27.0) |
| K10 ≥ 22 | 696 (22.7%) | 244 (27.1%) | 120 (41.0%) | 305 (37.4%) |

**Table S7:** Participant characteristics in 2017

|  | Working only | Studying and working | Studying only | NEET |
| --- | --- | --- | --- | --- |
| **Age group** |  |  |  |  |
| 18-25 | 1009 (33.2%) | 692 (70.4%) | 226 (68.8%) | 318 (41.6%) |
| 26-35 | 2029 (66.8%) | 292 (29.6%) | 102 (31.2%) | 448 (58.4%) |
| **Sex** |  |  |  |  |
| Male | 1642 (54.0%) | 481 (48.9%) | 151 (46.1%) | 283 (37.0%) |
| Female | 1396 (46.0%) | 503 (51.1%) | 177 (53.9%) | 483 (63.0%) |
| **IRSAD**, (mean, SD) | 6.0 (2.8) | 6.2 (2.9) | 6.2 (2.9) | 4.8 (3.0) |
| **Equivalised household income,** (mean, SD) | 69043.2 (33029.6) | 71223.5 (55839.5) | 52470.1 (30229.0) | 43463.5 (26897.9) |
| **Financial Hardship** | 592 (20.9%) | 220 (23.4%) | 78 (25.8%) | 254 (36.1%) |
| **CALD** | 736 (24.2%) | 256 (26.1%) | 82 (24.9%) | 211 (27.5%) |
| **Marital status: Married** | 1665 (54.8%) | 234 (23.7%) | 73 (22.2%) | 317 (41.4%) |
| **MHI-5** |  |  |  |  |
| Mean (SD) | 72.1 (17.7) | 71.7 (16.8) | 66.3 (18.6) | 63.6 (20.5) |
| Median (Q1, Q3) | 76.0 (60.0, 84.0) | 76.0 (64.0, 84.0) | 68.0 (56.0, 80.0) | 68.0 (48.0, 80.0) |
| MHI-5 < 52 | 459 (15.2%) | 151 (15.6%) | 80 (24.6%) | 247 (32.4%) |
| **K10 score** |  |  |  |  |
| Mean (SD) | 17.2 (7.1) | 17.2 (7.1) | 19.5 (7.8) | 20.4 (8.8) |
| Median (Q1, Q3) | 15.0 (12.0, 21.0) | 15.0 (12.0, 21.0) | 17.0 (13.5, 24.0) | 18.0 (13.0, 28.0) |
| K10 ≥ 22 | 677 (22.3%) | 236 (24.0%) | 108 (33.1%) | 286 (37.4%) |

**Table S8:** Participant characteristics in 2015

|  | Working only | Studying and working | Studying only | NEET |
| --- | --- | --- | --- | --- |
| **Age group** |  |  |  |  |
| 18-25 | 938 (33.3%) | 644 (66.8%) | 267 (69.2%) | 284 (37.9%) |
| 26-35 | 1878 (66.7%) | 320 (33.2%) | 119 (30.8%) | 466 (62.1%) |
| **Sex** |  |  |  |  |
| Male | 1530 (54.3%) | 474 (49.1%) | 144 (37.4%) | 312 (41.6%) |
| Female | 1287 (45.7%) | 491 (50.9%) | 242 (62.6%) | 438 (58.4%) |
| **IRSAD**, (mean, SD) | 5.9 (2.7) | 5.9 (2.9) | 6.4 (3.0) | 4.7 (2.9) |
| **Equivalised household income,** (mean, SD) | 64639.6 (33429.9) | 67417.2 (40320.6) | 55542.8 (60622.6) | 44664.4 (25891.1) |
| **Financial Hardship** | 690 (24.8%) | 229 (24.0%) | 134 (35.1%) | 291 (39.8%) |
| **CALD** | 779 (27.7%) | 245 (25.4%) | 120 (30.9%) | 215 (28.7%) |
| **Marital status: Married** | 1548 (55.0%) | 256 (26.6%) | 89 (23.0%) | 363 (48.4%) |
| **MHI-5** |  |  |  |  |
| Mean (SD) | 72.9 (16.4) | 72.9 (16.2) | 68.2 (19.9) | 66.8 (20.1) |
| Median (Q1, Q3) | 76.0 (64.0, 84.0) | 76.0 (60.0, 84.0) | 72.0 (56.0, 84.0) | 72.0 (56.0, 80.0) |
| MHI-5 < 52 | 405 (14.5%) | 125 (13.0%) | 83 (21.9%) | 173 (23.2%) |
| **K10 score** |  |  |  |  |
| Mean (SD) | 16.7 (6.6) | 17.2 (6.6) | 19.3 (8.3) | 19.9 (8.7) |
| Median (Q1, Q3) | 15.0 (12.0, 20.0) | 15.0 (12.0, 21.0) | 17.0 (13.0, 25.0) | 17.0 (13.0, 25.0) |
| K10 ≥ 22 | 517 (18.4%) | 219 (22.8%) | 133 (34.5%) | 267 (35.7%) |

**Table S9:** Participant characteristics in 2013

|  | Working only | Studying and working | Studying only | NEET |
| --- | --- | --- | --- | --- |
| **Age group** |  |  |  |  |
| 18-25 | 897 (32.6%) | 606 (66.4%) | 286 (73.9%) | 323 (39.5%) |
| 26-35 | 1855 (67.4%) | 306 (33.6%) | 101 (26.1%) | 496 (60.5%) |
| **Sex** |  |  |  |  |
| Male | 1507 (54.8%) | 469 (51.4%) | 177 (45.8%) | 283 (34.6%) |
| Female | 1244 (45.2%) | 444 (48.6%) | 210 (54.2%) | 536 (65.4%) |
| **IRSAD**, (mean, SD) | 5.6 (2.9) | 6.3 (2.7) | 6.0 (2.9) | 4.8 (2.9) |
| **Equivalised household income,** (mean, SD) | 63367.7 (34719.9) | 66643.4 (44346.8) | 49567.0 (32100.7) | 43268.5 (26736.9) |
| **Financial Hardship** | 631 (23.6%) | 219 (24.3%) | 108 (29.3%) | 368 (46.2%) |
| **CALD** | 820 (29.8%) | 246 (27.0%) | 164 (42.3%) | 244 (29.8%) |
| **Marital status: Married** | 1526 (55.5%) | 250 (27.3%) | 84 (21.7%) | 374 (45.7%) |
| **MHI-5** |  |  |  |  |
| Mean (SD) | 74.1 (16.5) | 74.5 (16.0) | 67.3 (18.7) | 69.6 (18.5) |
| Median (Q1, Q3) | 76.0 (68.0, 84.0) | 80.0 (68.0, 84.0) | 68.0 (56.0, 80.0) | 72.0 (58.0, 84.0) |
| MHI-5 < 52 | 371 (13.6%) | 122 (13.4%) | 86 (22.1%) | 169 (20.7%) |
| **K10 score** |  |  |  |  |
| Mean (SD) | 16.4 (6.2) | 16.3 (6.3) | 19.8 (7.7) | 18.3 (7.4) |
| Median (Q1, Q3) | 15.0 (12.0, 19.0) | 14.0 (12.0, 19.0) | 18.0 (13.0, 24.0) | 16.0 (13.0, 22.0) |
| K10 ≥ 22 | 469 (17.2%) | 161 (17.7%) | 138 (35.7%) | 221 (27.3%) |

**Table S10:** Participant characteristics in 2011

|  | Working only | Studying and working | Studying only | NEET |
| --- | --- | --- | --- | --- |
| **Age group** |  |  |  |  |
| 18-25 | 854 (32.1%) | 690 (67.2%) | 257 (72.9%) | 353 (44.6%) |
| 26-35 | 1855 (67.4%) | 306 (33.6%) | 101 (26.1%) | 496 (60.5%) |
| **Sex** |  |  |  |  |
| Male | 1503 (56.5%) | 538 (52.5%) | 148 (42.1%) | 253 (32.0%) |
| Female | 1159 (43.5%) | 487 (47.5%) | 204 (57.9%) | 537 (68.0%) |
| **IRSAD**, (mean, SD) | 5.9 (2.8) | 6.2 (2.8) | 5.5 (2.9) | 4.7 (3.0) |
| **Equivalised household income,** (mean, SD) | 58991.7 (28783.4) | 59855.3 (32943.0) | 42803.0 (26079.0) | 41093.9 (27968.4) |
| **Financial Hardship** | 742 (28.2%) | 305 (29.8%) | 119 (34.8%) | 370 (47.6%) |
| **CALD** | 693 (26.0%) | 317 (30.9%) | 161 (45.6%) | 255 (32.3%) |
| **Marital status: Married** | 1433 (53.9%) | 292 (28.4%) | 99 (28.2%) | 393 (49.7%) |
| **MHI-5** |  |  |  |  |
| Mean (SD) | 74.6 (15.9) | 73.2 (16.1) | 73.3 (16.5) | 67.8 (18.9) |
| Median (Q1, Q3) | 76.0 (64.0, 88.0) | 76.0 (64.0, 84.0) | 76.0 (64.0, 85.0) | 68.0 (56.0, 84.0) |
| MHI-5 < 52 | 282 (10.6%) | 123 (12.0%) | 35 (10.0%) | 174 (22.0%) |
| **K10 score** |  |  |  |  |
| Mean (SD) | 15.9 (5.9) | 16.4 (6.3) | 17.5 (7.0) | 18.9 (7.9) |
| Median (Q1, Q3) | 14.0 (12.0, 19.0) | 14.0 (12.0, 19.0) | 15.0 (12.0, 21.0) | 16.0 (13.0, 25.0) |
| K10 ≥ 22 | 408 (15.4%) | 166 (16.3%) | 82 (23.4%) | 244 (31.1%) |

**Table S11:** Participant characteristics in 2009

|  | Working only | Studying and working | Studying only | NEET |
| --- | --- | --- | --- | --- |
| **Age group** |  |  |  |  |
| 18-25 | 778 (36.4%) | 449 (71.3%) | 175 (68.2%) | 241 (40.4%) |
| 26-35 | 1359 (63.6%) | 180 (28.7%) | 82 (31.8%) | 355 (59.6%) |
| **Sex** |  |  |  |  |
| Male | 1194 (55.9%) | 289 (46.0%) | 124 (48.2%) | 198 (33.3%) |
| Female | 942 (44.1%) | 340 (54.0%) | 133 (51.8%) | 397 (66.7%) |
| **IRSAD**, (mean, SD) | 5.9 (2.8) | 6.6 (2.7) | 6.0 (3.0) | 4.4 (2.9) |
| **Equivalised household income,** (mean, SD) | 58519.9 (32554.1) | 58839.7 (30766.2) | 43779.0 (24326.2) | 37699.8 (25394.4) |
| **Financial Hardship** | 511 (24.2%) | 140 (22.8%) | 100 (40.0%) | 272 (48.0%) |
| **CALD** | 340 (15.9%) | 92 (14.6%) | 106 (41.4%) | 149 (25.1%) |
| **Marital status: Married** | 1124 (52.6%) | 163 (25.9%) | 78 (30.6%) | 282 (47.4%) |
| **MHI-5** |  |  |  |  |
| Mean (SD) | 74.5 (15.6) | 73.6 (15.4) | 68.6 (18.2) | 66.7 (19.2) |
| Median (Q1, Q3) | 80.0 (64.0, 84.0) | 76.0 (64.0, 84.0) | 72.0 (56.0, 84.0) | 68.0 (52.0, 84.0) |
| MHI-5 < 52 | 239 (11.3%) | 77 (12.4%) | 60 (23.7%) | 160 (27.1%) |
| **K10 score** |  |  |  |  |
| Mean (SD) | 15.7 (5.8) | 16.7 (6.8) | 17.6 (6.7) | 19.2 (8.1) |
| Median (Q1, Q3) | 14.0 (11.0, 18.0) | 15.0 (12.0, 19.0) | 16.0 (13.0, 20.0) | 17.0 (13.0, 25.0) |
| K10 ≥ 22 | 311 (14.6%) | 113 (18.1%) | 57 (22.4%) | 186 (31.7%) |

**Table S12:** Participant characteristics in 2007

|  | Working only | Studying and working | Studying only | NEET |
| --- | --- | --- | --- | --- |
| **Age group** |  |  |  |  |
| 18-25 | 895 (38.6%) | 392 (70.3%) | 111 (73.1%) | 208 (40.1%) |
| 26-35 | 1425 (61.4%) | 165 (29.7%) | 41 (26.9%) | 309 (59.9%) |
| **Sex** |  |  |  |  |
| Male | 1295 (55.8%) | 260 (46.6%) | 73 (47.9%) | 198 (33.3%) |
| Female | 1024 (44.2%) | 298 (53.4%) | 79 (52.1%) | 397 (66.7%) |
| **IRSAD**, (mean, SD) | 5.9 (2.9) | 6.3 (2.9) | 5.7 (3.1) | 4.6 (2.9) |
| **Equivalised household income,** (mean, SD) | 53107.1 (44114.8) | 49776.5 (25908.2) | 37706.2 (21255.9) | 33581.8 (20220.5) |
| **Financial Hardship** | 613 (27.0%) | 168 (30.7%) | 59 (39.6%) | 223 (44.6%) |
| **CALD** | 452 (19.5%) | 106 (19.0%) | 59 (38.7%) | 108 (20.9%) |
| **Marital status: Married** | 1120 (48.3%) | 147 (26.4%) | 37 (24.5%) | 270 (52.2%) |
| **MHI-5** |  |  |  |  |
| Mean (SD) | 73.9 (16.0) | 73.3 (14.9) | 68.4 (17.0) | 68.6 (20.9) |
| Median (Q1, Q3) | 76.0 (64.0, 84.0) | 76.0 (64.0, 84.0) | 72.0 (60.0, 80.0) | 75.0 (56.0, 84.0) |
| MHI-5 < 52 | 304 (13.1%) | 62 (11.1%) | 28 (18.4%) | 116 (22.4%) |
| **K10 score** |  |  |  |  |
| Mean (SD) | 16.2 (6.0) | 16.6 (5.8) | 17.9 (6.2) | 18.6 (8.4) |
| Median (Q1, Q3) | 14.0 (12.0, 19.0) | 15.0 (12.0, 20.0) | 17.0 (13.0, 21.0) | 15.0 (12.0, 23.0) |
| K10 ≥ 22 | 386 (16.9%) | 106 (19.1%) | 36 (24.1%) | 145 (28.6%) |
